# Combined Effects of *Ziziphus jujuba*, *Dimocarpus longan*, and *Lactuca sativa* on Sleep-Related Behaviors through GABAergic Signaling

**DOI:** 10.3390/foods13010001

**Published:** 2023-12-19

**Authors:** Gi Yeon Bae, Kayoung Ko, Eunseon Yang, Sung-Soo Park, Hyung Joo Suh, Ki-Bae Hong

**Affiliations:** 1 Department of Integrated Biomedical and Life Science, Graduate School, Korea University, Seoul 02841, Republic of Korea; rldus530@naver.com (G.Y.B.); suh1960@korea.ac.kr (H.J.S.); 2 Department of Food Science and Nutrition, Jeju National University, Jeju 63243, Republic of Korea; lv007@jejunu.ac.kr (K.K.); yes2789@jejunu.ac.kr (E.Y.); foodpark@jejunu.ac.kr (S.-S.P.); 3 BK21FOUR R&E Center for Learning Health Systems, Korea University, Seoul 02841, Republic of Korea

**Keywords:** botanical extract, sleep promotion, invertebrate, vertebrate, behavioral changes

## Abstract

We aimed to analyze the increase in the sleep-promoting effects based on the mixed ratio of botanical extracts, *Ziziphus jujuba* seeds, *Dimocarpus longan* fruits, and *Lactuca sativa* leaves, using animal models. Behavioral analyses, including an analysis of the total sleep time of *Drosophila melanogaster*, were conducted to select the optimal mixed ratio of the three botanical extracts. The effects were verified in a caffeine-induced sleepless model, specific neurotransmitter receptor antagonists, and ICR mice. In *D*. *melanogaster* exposed to 2.0% of each extract, group behavior was significantly reduced, and the mixed extracts of *Z*. *jujuba*, *D*. *longan*, and *L*. *sativa* (4:1:1 and 1:4:1) significantly increased the total sleep time with individual fruit flies. In the caffeine-induced insomnia model, mixed extracts (4:1:1 and 1:4:1) led to the highest increase in total sleep time. An analysis of locomotor ability revealed a significant reduction in the mobility percentage in the mixed extract groups (0:0:1, 1:0:1, 1:1:1, 4:1:1, and 1:4:1). The administration of *Z*. *jujuba* extract and mixed extracts (4:1:1) significantly increased the expression of GABA_A_-R, whereas the administration of the mixed extracts (4:1:1) and (1:4:1) significantly increased the expression of GABAB-_R_1 and GABAB-_R_2, respectively. *D*. *longan* extract and the mixed ratio (1:4:1) reduced the subjective nighttime movement and increased the total sleep time in the presence of flumazenil. An analysis of ICR mice indicated that the administration of mixed extracts (4:1:1) significantly increased sleep duration in a dose-dependent manner. These results indicated that the mixed ratio of *Z*. *jujuba*, *D*. *longan*, and *L*. *sativa* extracts, particularly the mixed ratio of 4:1:1, may have sleep-enhancing effects in fruit flies and mice. The study also identified changes in gene expression related to GABA receptors, indicating the potential mechanism for the observed sleep-promoting effects.

## 1. Introduction

Sleep is a vital physiological function crucial for maintaining human health and well-being. Both the quantity and quality of sleep are essential, and sleep disorders can be caused by numerous factors, such as physiological, psychological, environmental, and lifestyle-related elements. Persistent sleep imbalances adversely impact learning and memory, the immune system, cardiovascular health, and physical and mental well-being [1,2]. Insomnia, characterized by difficulty in regulating sleep latency and duration, is a common sleep disorder [3]. Although the current treatments for insomnia, including sedative–hypnotics and anxiolytics, lead to short-term improvement, they are associated with a risk of dependence, tolerance, withdrawal symptoms, side effects, cognitive and behavioral issues, and more difficulty in sleeping when used in the long term [4,5,6].

The use of natural products to relieve insomnia for mild or occasional insomnia offers advantages such as minimizing the risk of medication interaction and dependency. Herbal extracts such as valerian root, chamomile, passionflower, ashwagandha, and hops have shown potential benefits in managing sleep and insomnia through various experimental approaches [7,8,9,10,11,12]. These natural products and botanical remedies can be used individually or in combination to increase their efficacy. The mixed ratio is a crucial parameter that directly affects the efficiency and effectiveness of natural products. Achieving the optimal mixed ratio is essential due to factors such as selectivity, resource efficiency, cost-effectiveness, consistency, and reproducibility.

*Ziziphus jujuba* (jujube) seeds are traditional sleep-promoting natural products rich in alkaloids and saponin and have been used to treat sleep disorders, including insomnia, by regulating the nervous system related to gamma-aminobutyric acid (GABA) [13,14]. *Dimocarpus longan* fruits (longan) affect the activity of certain neurotransmitters in the brain that play a role in mood regulation and sleep–awake cycles and possess excellent anxiolytic and antioxidant properties; thus, they are a source of essential nutrients [15,16]. *Lactuca sativa* leaves (lettuce) contain bioactive compounds, including phytonutrients and antioxidants, and are a source of the predominant lactone that plays a role in muscle relaxation and the regulation of the sleep–wake cycle [16]. Although the water extracts of these natural products may have certain properties that could potentially contribute to sleep regulation, limited scientific evidence supporting their role in promoting sleep improvement based on mixed ratios exists.

*Drosophila melanogaster* is a model that has genetic and neurobiological similarities with mammals, including humans [17,18]. As their sleep patterns and molecular mechanisms that regulate sleep are known, their sleep behavior can be quantified and analyzed in a laboratory environment [19,20,21]. Genetic tools, such as transgenic flies and gene knockouts, aid in the exploration of the role of specific genes and neural circuits in sleep regulation by sleep-promoting active substances and in the identification of potential pharmaceuticals and non-pharmaceuticals [22]. To screen natural products with potential sleep-promoting properties and achieve the right balance of mixing ratios, *Drosophila* can help understand the sleep regulation of more complex organisms and the development of new treatments for sleep-related disorders.

In this study, we investigated the sleep-regulating properties and optimal mixed ratios of water extracts from three plant species—*Z. jujuba*, *D. longan*, and *L. sativa*—which are known for their potential involvement in sleep regulation. Utilizing a *Drosophila* model, we employed behavioral analysis to select the optimal extract concentration and mixed ratios that demonstrated sleep-modulating effects. Furthermore, we examined the expression profiles of specific neurotransmitter receptors associated with sleep initiation and maintenance and assessed the characteristics of the optimized mixed ratio using receptor antagonists. The efficacy of the selected extracts and ratios was subsequently validated through sleep-related behavioral analysis in ICR mice. This comprehensive investigation sheds light on the potential mechanisms underlying the sleep-promoting properties of mixed natural extracts and provides insights into their therapeutic applications for sleep-related disorders.

## 2. Material and Methods

### 2.1. Material Preparation

Water extracts obtained from *Z. jujuba*, *D. longan*, and *L. sativa* were provided by Serom Bio Co. (Gunpo, Republic of Korea). Dried seeds and leaves of Z. jujuba, D. longan, and L. sativa were purchased from Xi’an Sanjiang Bio-Engineering Co., Ltd. (Xi’an, Shaanxi Province, China) and extracted with hot water (*w*:*v* = 1:8), followed by reflux extraction at 120 ± 5 °C for 3 h. The extracts were filtered (Whatman No.2, What-man plc, Kent, UK), concentrated under reduced pressure at 65 ± 5 °C, and spray-dried (Inlet: 180 °C, Outlet: 100 °C). The powders obtained by extraction were maintained at −18 °C until further experiments and different batches were used to analyze the content of active compounds and sleep-related indicators. Propionic acid and tegosept (p-hydroxybenzoic acid methyl ester solution) were obtained from Daejung Chemicals (Siheung, Republic of Korea) and APExBIO (Houston, TX, USA). Flumazenil was purchased from Sigma-Aldrich (St. Louis, MO, USA) and pentobarbital sodium, Entobar, was purchased from Hanlim Pharmaceutical Co., (Yongin, Republic of Korea).

### 2.2. Total Polyphenol Content Assessment and Liquid Chromatography Analysis

Assessment of total polyphenol content was performed according to the modified Singleton method using gallic acid as standard [23]. The free amino acid and GABA contents of water extracts of *Z. jujuba* and *D. longan* were measured using the AccQ·Tag method (Bae et al., 2023). The high-performance liquid chromatography (HPLC) instrument used was a Waters product consisting of a 1525 pump (Binary HPLC Pump) and a 474 fluorescence detector (Scanning Fluorescence Detector). In addition, an ACCQ-Tag C18 column (3.9 mm × 150 mm I.D., 4 µm) was purchased from Waters and used, and the excitation wavelength of the fluorescence detector was set to 250 nm and the measurement wavelength was set to 390 nm. The flow rate was set at 1 mL per minute and 10 µL was injected. Mobile phase A was used by diluting 100 mL of AccuQTag Eluent A with 1 L of water, and mobile phase B was analyzed in gradient mode using 60% acetonitrile (water: acetonitrile, 40:60, *v*/*v*). The quercetin-3-glucuronide (Q3G) content of lettuce (*L. sativa*) water extract was detected at a wavelength of 350 nm using a YMC-Pack ODS-A column (250 mm × 4.6 mm, 5 μm). The mobile phase consisted of 0.5% formic acid in water (A) and 0.5% formic acid in acetonitrile (B). The gradient of the mobile phase was 80% A for 0 min, 77% A for 5 min, 73% A for 20 min, and 80% A for 25–30 min [24].

### 2.3. Experimental Animals

Wild-type *Drosophila melanogaster* Canton-S strain obtained from the Bloomington *Drosophila* Stock Center (Bloomington, IN, USA) was maintained at 24 ± 2 °C in 60 ± 5% relative humidity with a light:dark cycle of 12:12 and used for sleep-related behavior and gene expression analysis. Sucrose, agar, cornmeal, dried yeast, propionic acid, and p–hydroxybenzoic acid methyl ester solutions were used as the standard medium for *Drosophila* maintenance. Prior to sample exposure, 3–5-day-old male flies were collected under CO_2_ anesthesia. ICR mice (3-week-old, male) used in the pentobarbital-induced sleep test were purchased from Orient Bio (Seongnam, Republic of Korea). Animals were acclimated in an automatically managed condition room (12 h light:dark cycle, 45–55% relative humidity, at 22 ± 1 °C), and water and feed were supplied ad libitum. The pentobarbital-induced sleep test was performed with the approval of the Institutional Animal Care and Use Committee of Jeju National University (approval number: 2022-0037, approval date: 22 August 2022).

### 2.4. Behavioral Assays

Changes in activity within groups of flies following exposure to *Z. jujuba*, *D. longan*, and *L. sativa* extracts at different concentrations were assessed using a locomotor activity monitoring system (LAM, TriKinetics, Waltham, MA, USA). After placing 10 fruit flies in a transparent maintaining vial (diameter: 2.5 cm, length: 9.5 cm), the infrared detector was adjusted to focus at the center of the vial, and all measurements were recorded at every 30 min intervals. The concentration of each extract that induced a decrease in behavior was selected through the LAM system, and the changes in subjective nighttime and daytime activities and total sleep time of fruit flies stored in individual glass tubes according to each extract and extract mixed ratio were measured using the *Drosophila* Activity Monitoring system (DAM, TriKinetics). In addition, the DAM system was used to verify the effects of each extract and mixed ratio on the subjective nighttime and daytime activities and total sleep time in an insomnia model induced by 0.1% caffeine. Changes in behavior measured from the LAM and DAM systems were analyzed by data management software (TriKinetics) along with the control of environmental stimuli such as sound and light, and the provided text file data were visualized using Actogram J software. The subjective nighttime (22:01–10:00) and daytime (10:01–22:00) activities were calculated as the sum of all activity recorded during each 12 h period, and the total sleep time was calculated by summing min with 0 counts maintained for longer than 5 min in subjective nighttime phase [25]. Video tracking analysis was performed on adult fruit flies (male, 3 days) using 2.0% hot water extracts of *Z. jujuba*, *D. longan*, and *L. sativa* as a single treatment. The *Z. jujuba*: *D. longan*: *L. sativa* mixed extract (ratio 1:1:0, 1:0:1, 0:1:1, 1:1:1, 4:1:1, 1:4:1, 1:1:4/final concentration 2.0%) was administered for 7 days, and the results were analyzed. Video tracking analysis was conducted on adult fruit flies (male, 3 days) using a single treatment of 2.0% of *Z. jujuba*, *D. longan*, and *L. sativa* extracts and mixed extracts of *Z. jujuba*:*D. longan*:*L. sativa* (ratio 1:1:0, 1:0:1, 0:1:1, 1:1:1, 4:1:1, 1:4:1, 1:1:4/final concentration 2.0%) were administered for 7 days, and the results were analyzed. One fruit fly was placed in each of the nine circular arenas (8 mm in diameter and 0.1 mm in height), and the movement of the fruit flies was analyzed for 5 min using the EthoVision-XT system (Noldus Information Technology, Netherlands). For the behavioral analysis, we evaluated five items: distance moved, velocity, moving, not moving, and mobility. The total distance the fruit fly moved in the arena for 5 min was defined as the distance moved, and velocity was the speed at which the fruit fly moved. In addition, moving and not moving refer to the activity and inactivity of the fruit fly, and mobility refers to the pixel change value of spatial movement from the body point of the fruit fly [26].

### 2.5. Gene Expression

Adult fruit flies (male, 3 days) were exposed to the culture medium with a single concentration of 2.0% *Z. jujuba*, *D. longan*, and *L. sativa* extract and mixed extracts of *Z. jujuba*:*D. longan*:*L. sativa* (ratio 4:1:1, 1:4:1/final concentration 2.0%). After 7 days, fruit flies were placed in liquid nitrogen and then strongly vortexed to separate only the heads. Total RNA was extracted from the separated heads (100 heads per replicate) using TRIzol^®^ reagent (Invitrogen, Carlsbad, CA, USA). Quality-controlled total RNA samples were treated with RQ1 RNase-free DNase I (Promega, WI, USA), and 1 μg of total RNA was reverse transcribed using SuperScript^®^ III Reverse Transcriptase (Invitrogen). RpL32 (NM_001144655.3), an endogenous housekeeping gene, was used for result normalization using the ΔΔCt method [27]. Information on the target genes used in quantitative real-time polymerase chain reaction (qRT-PCR) with StepOne plus Software V. 2.0 (Applied Biosystems, CA, USA) is as follows: GABAA receptor (GABAA-R, NM_001274688. (1), GABAB receptor 1 (GABAB-R1, NM_001259104.2), GABAB receptor 2 (GABAB-R2, NM_001259104). (2), and 5-hydroxyryptophan receptor 1A (5HT1A, NM_166322.2).

### 2.6. Pentobarbital-Induced Sleep Test

To verify the sleep-promoting effects of the selected mixed ratio through behavioral and gene expression analysis using fruit flies, ICR mice were fasted for 20 h before the experiment, and the pentobarbital-induced sleep test was conducted between 1:00 pm and 5:00 pm. Forty minutes after the oral administration of mixed extracts of *Z. jujuba*:*D. longan*:*L. sativa* (ratio 4:1:1, 1:4:1/80 and 160 mg/kg), pentobarbital (42 mg/kg) was intraperitoneally injected, after which all mice were moved to an independent space, and sleep latency and total sleep time were measured [28].

### 2.7. Statistical Analysis

Data are presented as the mean ± standard error of the mean (SEM), and statistical analysis was performed using Prism (8.0.1., GraphPad Software Inc., San Diego, CA, USA). To compare groups, data were analyzed using Tukey’s multiple range test and Student’s *t*-test using a statistical package for social science (Version 25.0, IBM, Chicago, IL, USA). Statistical significance was set at *p* < 0.05.

## 3. Results and Discussion

### 3.1. Polyphenol Contents and Active Compounds of Z. jujuba, D. longan, and L. sativa Extracts

Differences in the total polyphenol content in each extract were investigated (Table 1). In addition, Table 1 and Supplementary Figure S1 show the contents and chromatograms of GABA, glycine, threonine, and Q3G, known as sleep-promoting substances, in the water extracts of *Z. jujuba*, *D. longan*, and *L. sativa*. In the case of total polyphenols, the content was highest in *L. sativa*, followed by *Z. jujuba* and *D. longan*. The water extract of Z. jujuba showed GABA, threonine, and glycine contents of 1.68, 0.73, and 0.08 mg/g, respectively. The water extract of D. longan showed a GABA content of 0.75 mg/g, which was higher than glycine (0.03 mg/g). The water extract of L. sativa contains 3.21 mg/g of Q3G, known as the active ingredient.

Sanjoinine A and jujuboside contained in seeds of *Z. jujuba* have been reported to have sleep-promoting activities by regulating the GABAergic system [29,30], but jujuboside (0.8 mg/g) [31] and pinosyn (1–0.4 mg/g) [32] contain a rather low content. GABA, which is reported to contain 333.37 to 150.31 μg/g in jujube seeds [33], is another active ingredient that promotes sleep. The water extract of *Z. jujuba* contained 1.68 mg/g of GABA, and 0.08 mg/g and 0.73 mg/g of glycine and threonine, respectively (Table 1). Glycine is a neurotransmitter that has a positive effect on sleep quality by preventing overstimulation. Glycine prevents the firing of orexin neurons, which regulate the sleep–wake cycle to promote sleep [34]. Threonine has been reported as a sleep-promoting molecule that can link the neuronal metabolism of amino acids to the GABAergic regulatory system in a *Drosophila* model [35]. *D. longan* fruit has a high amino acid content and contains 51 to 180 mg of GABA per 100 g of fresh material [36]. The water extract of *D. longan* contains 0.75 and 0.03 mg/g of GABA and Gly, respectively (Table 1), and appears to have sleep-promoting activity due to these amino acids. Lactucin, a known sleep-promoting substance, was not detected in the water extract of lettuce, and only Q3G, another active sleep-promoting substance, was contained at 3.21 mg/g (Table 1). Q3G promotes sleep by binding to GABAA-BDZ receptors [37,38]. Sleep promotion by water extracts of *Z. jujuba* and *D. longan* is presumed to be mainly due to GABA and the water extract of *L. sativa* seems to have shown sleep-promoting activity by Q3G.

### 3.2. Effects of Z. jujuba, D. longan, and L. sativa Extracts and Mixed Extracts on Sleep Behavior in Fruit Fly Groups and Individual Fruit Flies

**Figure 1:**
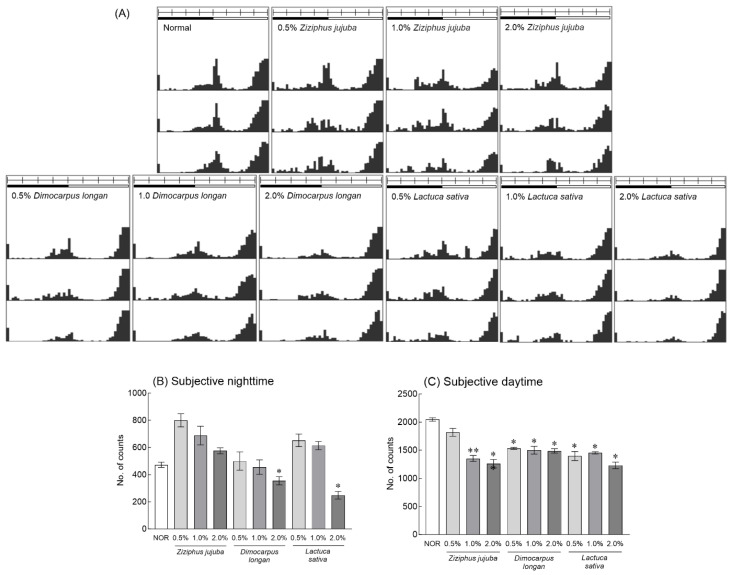
Effects of *Ziziphus jujuba*, *Dimocarpus longan*, and *Lactuca sativa* extracts on locomotor activity in fruit fly groups. This experiment was performed under constant darkness (DD) for 3 days (1 day: adaptation with normal diet; 3 days: experiment with treatments of extracts in sucrose agar media). Typical actograms of individual control flies (*n* = 50) and flies exposed to *Ziziphus jujuba* (*n* = 50), *Dimocarpus longan* (*n* = 50), and *Lactuca sativa* (*n* = 50) by dose. Average activity in a 30 min interval was calculated over 3 days. Black/white bars on the top of the actograms indicate dark (22:00 to 10:00) and light (10:00 to 22:00) phases. Activity during subjective nighttime (**A**), daytime (**B**) and total sleep time (**C**). Values are the means ± standard error of the mean (SEM) for each group. Symbols indicate significant differences at * *p* < 0.05 and ** *p* < 0.01.

**Figure 2:**
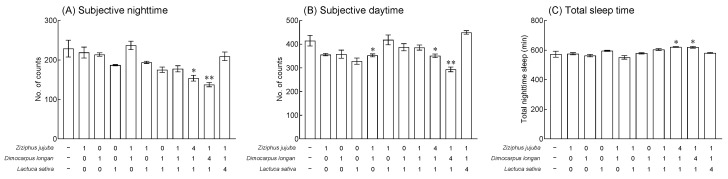
Effects of *Ziziphus jujuba*, *Dimocarpus longan*, and *Lactuca sativa* extracts and various mixed ratios on the sleep behavior of individual fruit flies. This experiment was performed under constant darkness (DD) for 5 days (1 day: adaptation with normal diet; 5 days: experiment with treatments of extracts in sucrose agar media). Typical actograms of individual control flies (*n* = 16) and flies exposed to *Ziziphus jujuba* (*n* = 16), *Dimocarpus longan* (*n* = 16), *Lactuca sativa* (*n* = 16), and each ratio (*n* = 16). Average activity in a 1 min interval was calculated over 5 days. Black/white bars on the top of the actograms indicate dark (22:00 to 10:00) and light (10:00 to 22:00) phases. Activity during subjective nighttime (**A**), daytime (**B**), and total sleeping time (**C**). Values are the means ± standard error of the mean (SEM) for each group. Symbols indicate significant differences at * *p* < 0.05 and ** *p* < 0.01.

Actograms were used to visualize the dose-dependent effects of *Z. jujuba*, *D. longan*, and *L. sativa* on the locomotor activity of fruit flies (Figure 1A). *Z.* *jujuba* extract did not significantly reduce locomotor activity in fruit flies at subjective nighttime compared to the normal group (NOR), but the behavior was reduced in a concentration-dependent manner (Figure 1B). The administration of 2% *D*. *longan* and *L*. *sativa* extracts significantly reduced the locomotor activity of fruit flies at subjective nighttime compared to the NOR group (Figure 1B, *p* < 0.05). In addition, the administration of 1.0% and 2.0% *Z*. *jujuba* extract and all concentrations of *D*. *longan* and *L*. *sativa* extracts significantly decreased the locomotor activity during subjective daytime compared to the NOR group (Figure 1C, *p* < 0.05 and *p* < 0.01, respectively). Based on the LAM system analysis, 2.0% of the selected *Z. jujuba*, *D. longan*, and *L. sativa* extracts and mixed extracts (ratio 1:1:0, 1:0:1, 0:1:1, 1:1:1, 4:1:1, 1:4:1, 1:1:4/final concentration 2.0%) were employed. The sleep-related parameters in fruit flies were evaluated based on the sum of total movements during subjective nighttime and daytime (No. of counts) and the sum of total sleep time (total nighttime sleep). When compared to the results from the fruit fly group through the LAM system, the behavior of an individual fruit fly exposed to 2.0% of *Z. jujuba*, *D. longan*, and *L. sativa* extracts in the DAM system showed no significant changes in the subjective nighttime and daytime activities and total sleep time compared to the NOR group (Figure 2). In the groups administered with mixed extract ratios of 4:1:1 and 1:4:1 of *Z. jujuba*, *D. longan*, and *L. sativa*, the subjective nighttime activity was significantly reduced compared to the NOR group (Figure 2A, *p* < 0.05 and *p* < 0.01). During the daytime, there was a significant decrease in the behavior of groups exposed to the 1:1 ratio of *Z. jujuba* and *D. longan* extracts and the mixed ratio of *Z. jujuba*, *D. longan*, and *L. sativa* extracts of 4:1:1 and 1:4:1 (Figure 2B, *p* < 0.05). The total sleep time showed a significant increase in the mixed ratio of *Z. jujuba*, *D. longan*, and *L. sativa* extracts in the group with 4:1:1 and 1:4:1 compared to the NOR group (Figure 2C, *p* < 0.05).

In this study, male *D*. *melanogaster* was used to investigate the changes in sleep-related parameters and locomotor activity following exposure to different concentrations of *Z*. *jujuba*, *D*. *longan*, and *L*. *sativa* and their combinations (Figure 1 and Figure 2). Although numerous studies have claimed beneficial synergistic interactions between natural product mixtures such as plant-based or plant-derived preparations, other studies have commented on antagonistic interactions between the components of the mixture [39,40]. Therefore, effective analyses and approaches are needed to identify synergistic combinations of natural product extracts and elucidate the mechanisms underlying their interactions. Radioligand binding assays, which focus on specific receptors in the central nervous system, can be used to assess the pharmacological activity of test compounds or botanical extracts that affect sleep regulation by evaluating their binding affinity [41]. While receptor binding analysis is a useful technique for identifying substances with potential sleep-promoting activity by evaluating binding affinity for neurotransmission-related receptors, it is part of a broader research process that also includes behavioral analysis, and electroencephalography is used to analyze binding only to specific receptors. *Drosophila* has been favored to analyze genetic interactions and neurophysiological-related molecular mechanisms; it is preferred as a model for sleep and circadian rhythm research, and it is evaluated as a valuable model to explore the sleep-promoting effects of natural product mixtures [42].

### 3.3. Effects of Z. jujuba, D. longan, and L. sativa Extracts and Mixed Extracts on Sleep Behavior and Locomotor Ability

**Figure 3:**
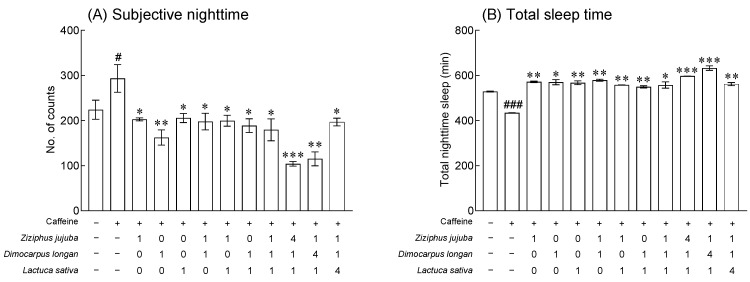
Effects of *Ziziphus jujuba*, *Dimocarpus longan*, and *Lactuca sativa* extracts and various mixed ratios on sleep behavior in a caffeine-induced insomnia model. This experiment was performed under constant darkness (DD) for 3 days (3 days: experiment with treatments of extracts in 0.1% caffeine with 5% sucrose agar media). Activity during subjective nighttime (**A**) and total sleeping time (**B**). Values are the means ± standard error of the mean (SEM) for each group. ^#^ *p* < 0.05 and ^###^ *p* < 0.001 vs. normal group; * *p* < 0.05, ** *p* < 0.01, and *** *p* < 0.001 vs. the 0.1% caffeine group.

**Figure 4:**
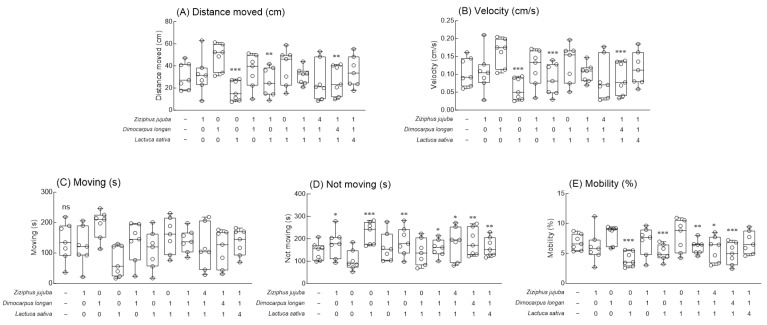
Effects of *Ziziphus jujuba*, *Dimocarpus longan*, and *Lactuca sativa* extracts and various mixed ratios on (**A**) distance moved, (**B**) velocity, (**C**) moving, (**D**) not moving, and (**E**) mobility of fruit flies during the 5 min observation period in the open field assay. After 7 days of feeding, the locomotion during the 5 min observation period in the video tracking was analyzed using the EthoVision-XT system. Values are the means ± standard error of the mean (SEM) for each group. Symbols indicate significant differences at * *p* < 0.05, ** *p* < 0.01, and *** *p* < 0.001. ns, not significant.

Compared to the normal group, in the group administered 0.1% caffeine (caffeine-induced insomnia model), movement at night was significantly increased, and total sleep time was decreased (Figure 3, *p* < 0.05 and *p* < 0.01). In addition, 2.0% *Z. jujuba*, *D. longan*, and *L. sativa* extracts reduced the activity at subjective nighttime to the level of the normal group compared to the 0.1% caffeine group, and the reduction was particularly high in the *D. longan* extract (Figure 3A, *p* < 0.05 and *p* < 0.01). In addition, all mixed extract groups of *Z. jujuba*, *D. longan*, and *L. sativa* extracts significantly reduced subjective nighttime activity compared to the 0.1% caffeine group (Figure 3A, *p* < 0.05), and the mixed ratios 4:1:1 and 1:4:1 showed the highest reduction (Figure 3A, *p* < 0.01 and *p* < 0.001). In the case of total sleep time, the 2.0% *Z. jujuba*, *D. longan*, and *L. sativa* extract significantly increased the total sleep time compared to the 0.1% caffeine group (Figure 3B, *p* < 0.05 and *p* < 0.01). The mixed extracts significantly increased the total sleep time when compared to the 0.1% caffeine group in all mixed ratios (Figure 3B, *p* < 0.05, *p* < 0.01, and *p* < 0.001), and the mixed ratio of *Z. jujuba*, *D. longan*, and *L. sativa* extract, 4:1:1, and 1:4:1, showed significantly higher sleep time. An analysis of the total movement and total sleep time at subjective nighttime revealed that the mixed ratio containing *Z. jujuba* and *D. longan* led to a high rate of activity. The distances moved (mm) and velocities (mm/s) of the groups exposed to a 2.0% *L. sativa* extract and mixed extracts of 1:0:1 and 1:4:1 for 7 days were significantly reduced compared to those of the normal group (Figure 4A,B. *p* < 0.01 and *p* < 0.001). No significant change in moving (sec) was observed in the groups exposed to a single extract and mixed extracts, but a significant increase was observed in terms of not moving (sec) when the groups were exposed to 2.0% *L. sativa* extract and mixed ratios of 1:0:1, 1:1:1, 4:1:1, 1:4:1, and 1:1:4 (Figure 4D, *p* < 0.05, *p* < 0.01, and *p* < 0.001). In the results related to mobility (%), no significant changes were observed in the groups exposed to *Z. jujuba*, *D. longan*, and *L. sativa* compared to the normal group, but the mobility was significantly decreased in the groups exposed to the mixed ratios of 0:0:1, 1:0:1, 1:1:1, 4:1:1, and 1:4:1 (Figure 4E, *p* < 0.05, *p* < 0.01, and *p* < 0.001).

Using a caffeine-induced insomnia model and video tracking analysis, we investigated that the mixed extracts of *Z*. *jujuba*, *D*. *longan*, and *L*. *sativa* alleviated the behavior altered by 0.1% caffeine (Figure 3) and regulated the locomotor abilities such as distance moved, velocity, and mobility (Figure 4). In a previous study, we performed a behavioral analysis of *D*. *melanogaster* to verify the concentration of GABA, an inhibitory neurotransmitter, and 5-hydroxytryptophan (5-HTP), a precursor of serotonin, which affect locomotor activity and sleep time. We showed that the GABA/5-HTP mixture showed a synergistic sleep-promoting effect when compared to a single amino acid [43]. Moreover, sleep-related behavior measurements in fruit flies identified hop varieties with excellent sleep-promoting effects, including cascade, hallertau, and saaz, and that total sleep time at subjective nighttime was improved compared to exposure to single extracts when mixed with valerian extracts in both baseline and caffeine-treated conditions [44]. Inoue et al. developed an automated sleep and rhythm analysis system and reported the effect of Japanese traditional herbal medicines on caffeine-induced insomnia in *Drosophila* [45]. Additionally, Ko reported that a six-herb mixture of an anti-insomnia formula can effectively attenuate caffeine-induced arousal in a fruit fly model and change the sleep time by affecting the tetrameric potassium channel through a short-sleep mutant fly line [46].

### 3.4. Effects of Z. jujuba, D. longan, and L. sativa Extracts and Mixed Extracts on Gene Expression and Flumazenil-Induced Behavioral Changes

**Figure 5:**
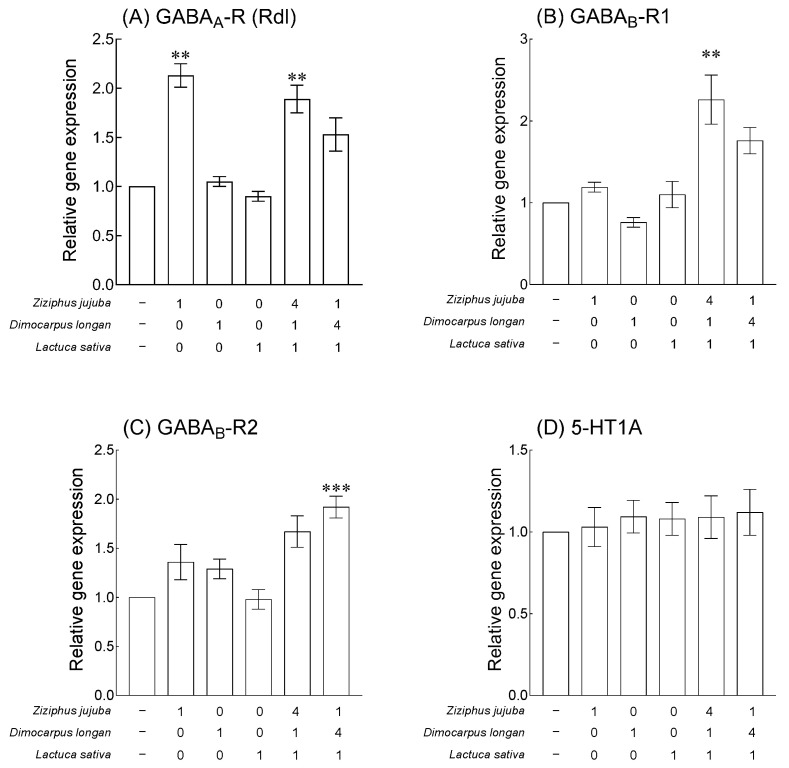
Effects of *Ziziphus jujuba*, *Dimocarpus longan*, and *Lactuca sativa* extracts and various mixed ratios on GABA_A_-R (Rdl), GABA_B_-R1, GABA_B_-R2, and 5-HT1A gene expression in fruit flies. Values are the means ± standard error of the mean (SEM) from 150 fruit flies per group. Symbols indicate significant differences at ** *p* < 0.01 and *** *p* < 0.001.

**Figure 6:**
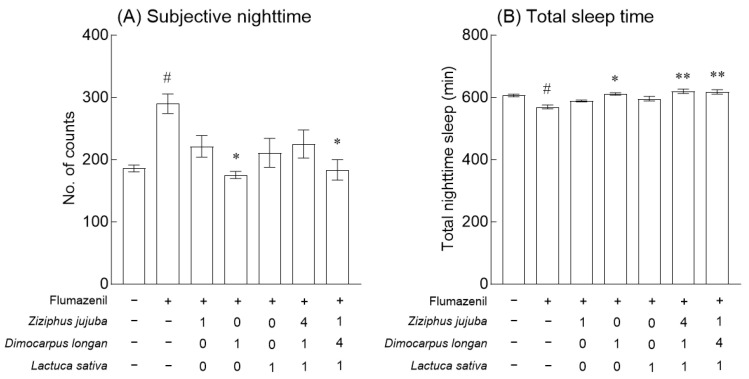
Effects of *Ziziphus jujuba*, *Dimocarpus longan*, and *Lactuca sativa* extracts and various mixed ratios on locomotor activity in flumazenil-treated fruit flies. This experiment was performed under constant darkness (DD) for 3 days (3 days: experiment with treatments of extracts in 0.01% flumazenil with 5% sucrose agar media). Activity during subjective nighttime (**A**) and total sleeping time (**B**). Values are the means ± standard error of the mean (SEM) for each group. Symbols indicate significant differences at * *p* < 0.05 and ** *p* < 0.01. ^#^ *p* < 0.05 vs. normal group; * *p* < 0.05, ** *p* < 0.01, and ** *p* < 0.01 vs. the 0.01% flumazenil group.

Based on the results of behavioral analysis using fruit flies and the caffeine-induced insomnia model, the optimal mixed ratio of *Z. jujuba*, *D. longan*, and *L. sativa* extracts considered was 4:1:1 and 1:4:1. The expression of neurotransmitter-related receptors was analyzed after 7 days of exposure. Compared with the expression of *GABA_A_-*R in the normal group, it was significantly increased in the groups exposed to *Z. jujuba* extract and mixed extracts (4:1:1) (Figure 5A, *p* < 0.01). In addition, the expression of *GABA_B_-R1* was significantly increased in the groups exposed to the mixed extracts (4:1:1) compared to the normal group (Figure 5B, *p* < 0.01), and the mixed extracts (1:4:1) significantly increased the expression of *GABA_B_-R2* (Figure 5C, *p* < 0.001). In the case of the expression of gene *5-HT1A*, no significant difference was observed among the single extract and mixed extract groups. Based on the increase in the expression of GABA receptors, changes in behavior were analyzed using Flumazenil, a benzodiazepine antagonist, to analyze the GABA receptor binding capacity of *Z. jujuba*, *D. longan*, and *L. sativa* extracts and mixed extracts (Figure 6). When compared to the normal group, exposure to 0.01% flumazenil significantly regulated the movement and total sleep time at subjective nighttime (Figure 6A,B, *p* < 0.05). The group exposed to 2% *D. longan* extract along with flumazenil significantly lowered the subjective nighttime movements and increased the total sleep time compared to the group exposed to 0.01% flumazenil (Figure 6A,B, *p* < 0.05). Compared to the group exposed to 0.01%, in the mixed extract (1:4:1) group of *Z. jujuba*, *D. longan*, and *L. sativa* (Figure 6A, *p* < 0.05), the subjective nighttime movement was significantly reduced, and the total sleep time was significantly increased in both mixed material (1:4:1, 4:1:1) groups (Figure 6B, *p* < 0.01).

**Figure 7:**
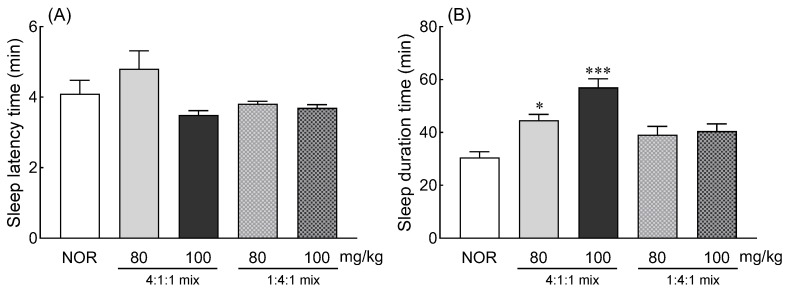
Effects of *Ziziphus jujuba*, *Dimocarpus longan*, and *Lactuca sativa* extracts on (**A**) sleep latency time and (**B**) sleep duration in ICR mice that were intraperitoneally administered pentobarbital (42 mg/kg). Values are the means ± standard error of the mean (SEM) for each group. Different letters indicate significant differences at *p* < 0.05 by Tukey’s test. * *p <* 0.05 and *** *p <* 0.001 vs. BDZ using Student’s *t*-test. NOR (normal, 0.9% saline), BDZ (Benzodiazepine 0.2 mg/kg).

Our results indicated that the mixed extracts of *Z*. *jujuba*, *D*. *longan*, and *L*. *sativa* enhanced the mRNA expression of inhibitory neurotransmission-related receptor genes in the brain (Figure 5) and verified the target receptor of the exposed treatment by behavioral analysis using the antagonist of GABA receptors (Figure 6). Moreover, the results obtained from the pentobarbital-induced sleep test verified that the administration of mixed extracts of *Z*. *jujuba*, *D*. *longan*, and *L*. *sativa* increased sleep duration (Figure 7). Jujuboside A and GABA, the main active components of *Z*. *jujuba* seeds, exert a sleep-regulating effect by significantly increasing the transcription level of GABA receptors in the cultured hippocampal neurons of rats [33,47]. In addition, the seeds of *Z*. *jujuba*, extracted with 50% ethanol after removing the lipid fraction, contain a total of 35 phytochemicals and interact with 71 anxiolytic targets, especially metabolites produced by gut microbes such as C-glycosides and jujubosides, which are involved in the regulation of serotoninergic and GABAergic synaptic pathways [48]. In *D*. *longan* fruits, the content of free amino acids, including GABA, accounts for 62.28–92.05% of the total amino acids, and *Longanae arillus* extract reduces sleep latency and extends sleep time [15,16]. *L*. *sativa* extract has been reported to enhance pentobarbital hypnosis without major toxic effects, and behavior analysis, electroencephalography, and GABA_A_ receptor binding analysis have demonstrated that the active ingredient, Q3G, has sleep-inducing and non-rapid eye movement and sleep-improving effects [38,49,50]. In addition, numerous double-blind randomized placebo-controlled trials indicated that *L*. *sativa* seed extracts improved the effect on insomnia related to disease and pregnancy [51,52]. In summary, the correlation between the results reported to date and this study is that the main components of the three plant extracts bind to the GABA_A_ receptor and regulate the gene expression of *GABA*-related receptors to improve the total sleep time.

### 3.5. Effects of Z. jujuba, D. longan, and L. sativa Extracts and Mixed Extracts on Pentobarbital-Induced Sleeping Behaviors

The effects of exposure to *Z. jujuba*, *D. longan*, and *L. sativa* extracts and mixed extracts on changes in sleep-related behavior in fruit flies were validated using ICR-mice, a vertebrate animal (Figure 7). Two mixing ratios (1:4:1, 4:1:1) were provided with the total volume being 80 and 100 mg/kg. The administration of 80 and 100 mg/kg of the two mixing ratios did not induce significant changes in sleep latency time compared to the normal group (Figure 7A). However, compared to the normal group, the administration of a mixed ratio of 4:1:1 with a high content of *Z. jujuba* increased the sleep duration time in a concentration-dependent manner (Figure 7A, *p* < 0.05 and *p* < 0.001).

Sleep tests utilizing barbiturates such as sodium thiopental and pentobarbital have been used to analyze the effects of food ingredients and natural products to treat complex chronic diseases or symptoms such as insomnia [15,53]. Among traditional plant extracts used to treat insomnia, *Ulmus macrocarpa*, *Rosa multiflora*, nutmeg, gardenia, and *Coptis chinesis* were materials with insufficient scientific evidence. By screening through a pentobarbital-induced sleep test in a mouse model, it was reported that *R*. *multiflora* is involved in changes in sleep latency and duration [54]. In addition, in the case of *Z. jujuba* seeds and walnut peptide, it was reported that the combined use of GABA improves sleep quantity by inducing changes in neurotransmission through pentobarbital-induced sleep test, molecular biological techniques, and ultra-high performance liquid chromatography–mass spectrometry analysis [55,56]. In addition, the analysis of sleep latency and duration reported differences in sleep-promoting effects depending on the type of lettuce and is also used as a tool to verify expected active ingredients [38,50]. An analysis of changes in behavior and gene expression in fruit flies and the use of behavioral pharmacology methods in mice can be used as a method to select and verify the mixing ratio of various materials in addition to screening the sleep-promoting effect of materials.

## 4. Conclusions

Research on the sleep-promoting effects of botanical extracts and their mixed ratios is crucial for several reasons, such as the variability in plant compounds, combination synergy, and long-term safety and efficacy. Because of the complex composition of plant extracts, including phytochemicals such as alkaloids, flavonoids, and terpenes, changes in sleep-promoting properties depending on the mixed ratio must be examined. In addition, research using animal models is essential to explore the sleep-promoting effects of plant extract-based treatments, identify effective combinations and ratios, and understand the underlying mechanisms of efficacy. Our findings provide compelling evidence that the mixed ratio of *Z*. *jujuba*, *D*. *longan*, and *L*. *sativa* extracts, particularly in the ratio of 4:1:1, exhibits promising sleep-enhancing properties in both fruit flies and mice. The observed changes in gene expression related to GABA receptors offer insights into the potential mechanisms underlying these effects. Further research using electrophysiology may help elucidate the therapeutic applications of these mixed botanical extracts for sleep-related disorders in humans and provide scientific evidence for developing functional food formulations.

## Figures and Tables

**Table 1:** Contents of total polyphenols and active compounds in water extracts of *Z. jujuba*, *D. longan*, and *L. sativa*.

Water Extract	Polyphenol	GABA	Threonine	Glycine	Q3G
	(mg/g)				
*Z. jujuba*	238.19 ± 7.16	1.68 ± 0.09	0.73 ± 0.04	0.08 ± 0.00	-
*D. longan*	177.00 ± 4.39	0.75 ± 0.01	-	0.03 ± 0.00	-
*L. sativa*	286.65 ± 4.38	-	-	-	3.21 ± 0.23

Values are the means ± standard deviation (SD) for each group. GABA, gamma-aminobutyric acid; Q3G, quercetin-3-glucuronide; -, not detected.

## Data Availability

Data are contained within the article and Supplementary Materials.

## Supplementary Materials

The following supporting information can be downloaded at: https://www.mdpi.com/article/10.3390/foods13010001/s1, Figure S1: High-performance liquid chromatography of glycine, threonine, GABA and in the water extract of *L. sativa*, *Z. jujuba* and *D. longan*. GABA, gamma-aminobutyric acid; Q3G, quercetin-3-glucuronide.
