# Supplementary material for: Combined Effects of Ziziphus jujuba, Dimocarpus longan, and Lactuca sativa on Sleep-Related Behaviors through GABAergic Signaling
Source: Foods. 2023 Dec 19;13(1):1. doi: 10.3390/foods13010001 (PMC10778002; doi:10.3390/foods13010001)
Supplement: Supplementary file 1 [file foods-13-00001-s001.zip › foods-2737266-supplementary.pdf]

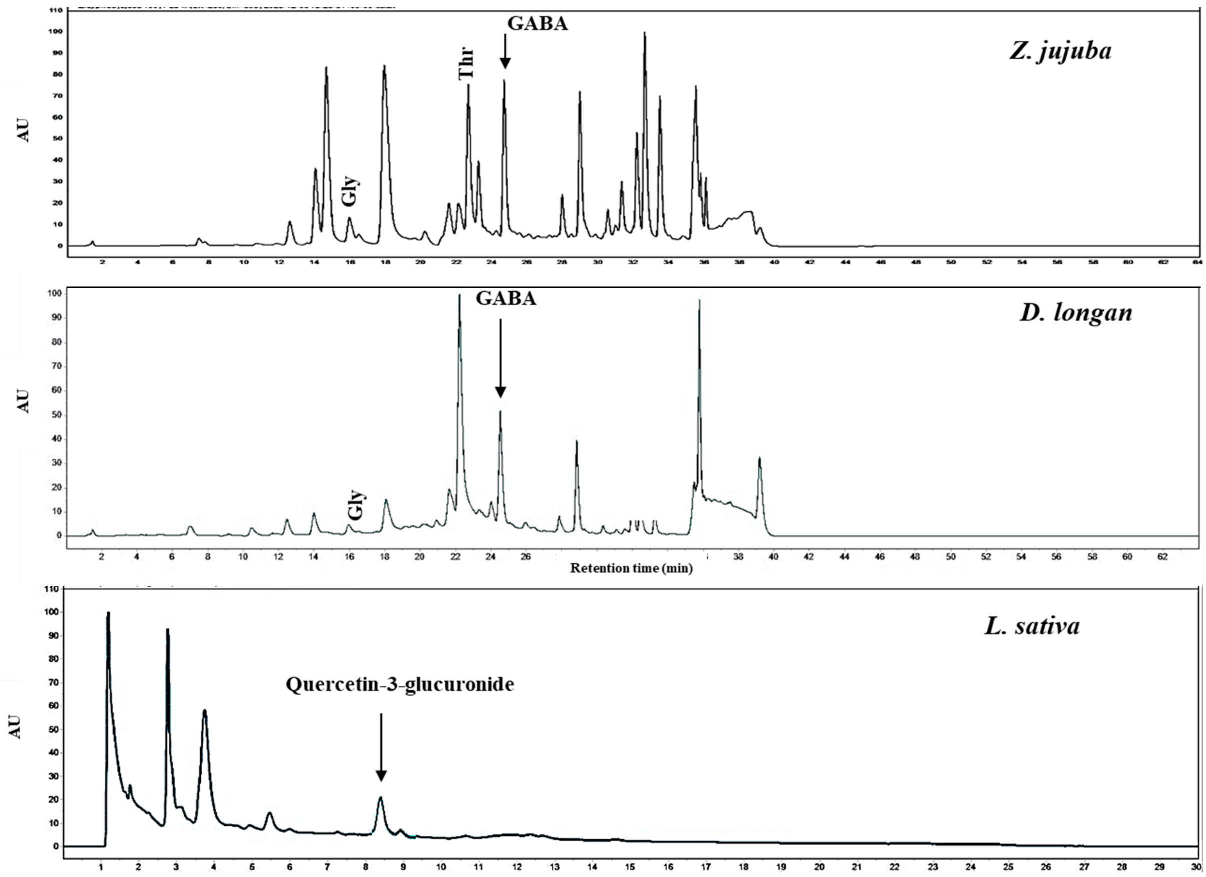

Supplementary Figure S1. High-performance liquid chromatography of glycine, threonine, GABA and in the water extract of *L. sativa, Z. jujuba* and *D. longan*. GABA, gamma-aminobutyric acid; Q3G, quercetin-3-glucuronide
